# Self Reported Incidence and Morbidity of Acute Respiratory Illness among Deployed U.S. Military in Iraq and Afghanistan

**DOI:** 10.1371/journal.pone.0006177

**Published:** 2009-07-08

**Authors:** Bryony W. Soltis, John W. Sanders, Shannon D. Putnam, David R. Tribble, Mark S. Riddle

**Affiliations:** 1 Department of Preventive Medicine, Uniformed Services University of Health Sciences, Bethesda, Maryland, United States of America; 2 Naval Medical Research Center Detachment, Lima, Peru; 3 U. S. Naval Medical Research Unit No. 2, Jakarta, Indonesia; 4 Infectious Disease Clinical Research Program, Uniformed Services University of the Health Sciences, Bethesda, Maryland, United States of America; 5 Enteric Diseases Department, Naval Medical Research Center, Silver Spring, Maryland, United States of America; Cincinnati Children's Hospital Medical Center, United States of America

## Abstract

**Background:**

Historically, respiratory infections have had a significant impact on U.S. military missions. Deployed troops are particularly at high risk due to close living conditions, stressful work environments and increased exposure to pathogens. To date, there are limited data available on acute respiratory illness (ARI) among troops deployed in support of ongoing military operations, specifically Operation Enduring Freedom (OEF) and Operation Iraqi Freedom (OIF).

**Methods:**

Using self-report data from two sources collected from troops deployed to Iraq, Afghanistan and the surrounding region, we analyzed incidence and risk factors for ARI. Military personnel on mid-deployment Rest & Recuperation (R&R) or during redeployment were eligible to participate in the voluntary self-report survey.

**Results:**

Overall, 39.5% reported having at least one ARI. Of these, 18.5% sought medical care and 33.8% reported having decreased job performance. The rate of self-reported ARI was 15 episodes per 100 person-months among those taking the voluntary survey, and 24.7 episodes per 100 person-months among those taking the clinic health questionnaire. Negative binomial regression analysis found female sex, Navy branch of service and lack of flush toilets to be independently associated with increased rates of ARI. Deployment to OIF, increasing age and higher rank were also positively associated with ARI risk.

**Conclusions:**

The overall percentage of deployed military personnel reporting at least one acute respiratory illness decreased since earlier parts of OIF/OEF. However, the reported effect on job performance increased tremendously. The most important factors associated with increased respiratory infection are female sex, Navy branch of service, lack of improved latrine facilities, deployment to OIF, increasing age and higher rank.

## Introduction

Respiratory illness is one of the most common causes of acute infectious disease among adults in the U.S. [1], [2]. Military personnel are at particularly high risk for respiratory infections because of close living conditions, stressful work environments and exposure to novel pathogens in disease-endemic areas during deployments [2], [3]. Non-battle injuries and illness have been responsible for more lost work-time and hospitalizations than combat injuries in U.S. military history [4], [5]. Respiratory illnesses in particular have had a significant impact on military missions [4]–[6]. Historically, influenza outbreaks have seriously impacted unit readiness [2], [7]. However, even relatively minor respiratory infections can impact operational performance [7]. For example, common-cold type respiratory symptoms were widespread and a frequent cause of minor morbidity during Operations Desert Shield and Desert Storm [8]. This was especially true during the initial deployment phase and crowding, as well as during periods of new unit movement into theater (8).

The U.S. currently has more than 200,000 military personnel deployed to Southwest/South central Asia [9]. Respiratory illness may cause a significant increase in health care utilization, as well as potential deployed personnel shortfalls. It is imperative that medical providers and policy makers understand the incidence and impact of respiratory illness in ongoing military operations. A recent study showed 69.1% of persons responding reported having a respiratory illness during their deployment to Iraq or Afghanistan [4], with 17% reported seeking medical attention [4]. This rate is considerably higher compared to an expected acute respiratory illness rate of ∼10% for those seeking medical attention among a military population in garrison (at home base) for a similar time period in the United States [10]. As there are scarce data beyond these currently available, it is important to further define the risk factors associated with acquiring respiratory illnesses, as well as the impact they have on the individual and on the mission.

This study provides current information on the incidence and impact of acute respiratory illness among deployed military personnel during 2005–2006. We also describe associations between respiratory illness and demographic factors, as well as characterize the seasonal distribution of self-reported respiratory illness. The information gained from this study can inform health-care providers and medical planners in hopes of improving management and prevention of respiratory illness among deployed troops. Ultimately, this will improve U.S. military operational readiness and efficiency.

## Results

### Anonymous Mid-, Post-deployment Questionnaire

From January 2005 through January 2006, at total of 2,872 deployed military troops completed and returned the questionnaire. Of these, 86.0% (n = 2,450) were Army personnel, 6.6% (n = 187) were Marine Corps personnel, 5.0% (n = 141) were Navy and 2.0% (n = 56) were Air Force personnel. The majority of the population belonged to command or support units (as opposed to combat units), 52.4% (n = 1,479) and 52.3% (n = 1,477) were Reserve or National Guard troops. Of troops responding, 71.4% (n = 2,015) were deployed in support of OIF, while 18.2% (n = 513) were deployed to OEF and 26.2% (n = 739) had deployed in support or OIF or OEF previously. Of those responding, 68.5% (n = 1,885) reported having flush toilets, while 31.5% (n = 866) reported using chemical/burn or bag/trench latrines. Nearly a third of respondents (32.6%) reported smoking at least ½ pack per day of cigarettes (Table 1).

**Table 1:** Demographic characteristics of 2,872 deployed U.S. military personnel who completed the voluntary respiratory infection questionnaire Jan 2005 to Jan 2006.

Characteristic	Frequency	Percent
Age in years, mean (range)	29.1	(19–59)
Gender		
Male	2,473	86.8
Female	339	13.2
Rank		
E1–E4	1,446	50.9
E5–E6	926	32.6
E7–E9	210	7.4
Officer/CWO	261	9.2
Duty Status		
Active duty	1,346	47.7
Reserve/Guard	1,477	52.3
Operation		
Iraqi Freedom	2,015	71.4
Enduring Freedom	513	18.2
Both	271	9.6
Neither	25	0.9
Branch		
Army	2,450	86.0
Air Force	56	2.0
Marine Corps	187	6.6
Navy	141	5.0
Other	16	0.6
Unit type		
Command/support	1,479	52.4
Ground	961	34.1
Other	382	13.5
Prior deployment	739	26.2
Latrine type		
Flush	1,885	68.5
Chemical/burn	832	30.2
Bag/trench/other	34	1.2
Cigarette smoking		
None	1,782	67.4
1/2 pack/day	436	16.5
1 pack/day	333	12.6
>1 pack/day	92	3.5
Questionnaire site		
Kuwait	342	12.0
Qatar	2,434	85.3
Turkey	79	2.8
Days in theater, mean (range)	164	(1, 826)

Overall, 39.5% (n = 1,128) of troops reported having at least one respiratory infection during the deployment with 20.2% reporting more than one respiratory infection. Duration of illness was between 4 to 7 days in 61.7% of troops reporting an illness. Of those who reported a respiratory infection, 29.4% also reported having a fever, and 18.5% sought medical care. Of those reporting respiratory infections, 33.8% (n = 377) reported the respiratory illness decreased job performance. Fourteen percent (n = 155) of those with self-reported respiratory infections were confined to quarters (given bed rest) or put on light duty (restricted work) and 1.2% (n = 13) reported requiring hospitalization due to the respiratory infection.

Based on self-reported disease and time-in-theater, the overall rate of respiratory infection was 15.0 (95% CI 14.3–15.6) episodes per 100 person-months. The rate of attending sick call for a respiratory infection was 5.1 (95% CI 4.8–5.5) visits per 100 person-months, and the overall rate of troops being confined to quarters was 2.4 (95% CI 2.2–2.7) cases per 100 person-months. The rate of hospital days among those reporting respiratory infections was 0.12 (95% CI 0.07–0.19) days per 100 person-months.

A multivariable negative binomial regression model for self-reported respiratory illness and covariates of military branch (Navy compared to all others), rank (categorical, junior enlisted referent), female sex, deployment to OIF, latrine type (flush toilet referent) and age was fit. (Table 2) Navy branch (IRR 1.81, p<0.001), non-commissioned/commissioned officers (E7–E9 IRR 1.47, p = 0.01; Officer/Chief Warrant Officer IRR 1.75, p<0.001), female sex (IRR 1.33, p = 0.004) and use of chemical/burn (IRR 1.50, p<0.001) were significantly associated with increased rates of self-reported respiratory illness. After adjusting for the other factors, deployment to OIF trended towards and association with increased respiratory rates (IRR 1.12, 95% CI 0.96–1.30), as did increase age (IRR 1.01 per year, p = 0.07) and tobacco use of one pack per day (IRR 1.19, 96%CI 0.96–1.46).

**Table 2:** Multivariate negative binomial regression of the association between rate of self-reported respiratory infection and covariates, voluntary respiratory questionnaire data Jan 2005 to Jan 2006.

Covariate	IRR	(95% CI)	P-value
Branch			
Army, Air Force, Marines	Reference		
Navy	1.81	(1.36–2.40)	<0.001
Rank			
E1–E4	Reference		
E5–E6	1.18	(0.99–1.39)	0.06
E7–E9	1.47	(1.09–1.98)	0.01
Officer/CWO	1.75	(1.36–2.25)	<0.001
Sex			
Male	Reference		
Female	1.33	(1.10–1.61)	0.004
Operation			
Enduring Freedom/Other	Reference		
Iraqi Freedom	1.12	(0.96–1.30)	0.14
Latrine type			
Flush	Reference		
Chemical/burn	1.50	(1.30–1.73)	<0.001
Bag/trench/other	1.66	(0.89–3.10)	0.1
Per year of age increase	1.01	(0.99–1.02)	0.07
Tobacco Use (smoking)			
None	Reference		
1/2 pack per day (PPD)	1.10	(0.91–1.33)	0.3
1 PPD	1.19	(0.96–1.46)	0.1
More than 1 PPD	1.16	(0.80–1.68)	0.4

### Health Screening Form

A total of 15,463 troops completed the Troop Medical Clinic health screening form between February 2005 and February 2006. Of these, 72.3% (n = 11,175) were deployed to Iraq, and 15.6% (n = 2,420) were serving in Afghanistan. The remainder of the troops were from Kuwait (n = 1,584) or other countries in the region (n = 57). Most of the troops completing the questionnaire were enlisted personnel in the ranks of E4 – E6 (70.5%) and male (86.3%) which matched the generalized demographics of deployed troops. Officers and warrant officers accounted for 8.4% (n = 1,262) of the population (Table 3).

**Table 3:** Demographic characteristics of deployed U.S. military personnel who completed the Qatar Clinic health screening questionnaire Feb 2005 to Feb 2006, N = 15,463*.

Characteristic	Frequency, n	Percent
Mean age, years (range)	28.6	(19–54)
Sex		
Male	13,170	86.3
Female	2,090	13.7
Rank		
E1–E3	2,199	14.7
E4–E6	10,547	70.5
E7–E9	946	6.3
O1–O3	652	4.4
O4–O6	488	3.3
Chief Warrant Officer	122	0.8
Country of deployment		
Afghanistan	2,420	15.7
Iraq	11,175	72.3
Kuwait	1,584	10.2
Other	57	0.4

* denominator for characteristic varies due to missing values.

**Figure 1:**
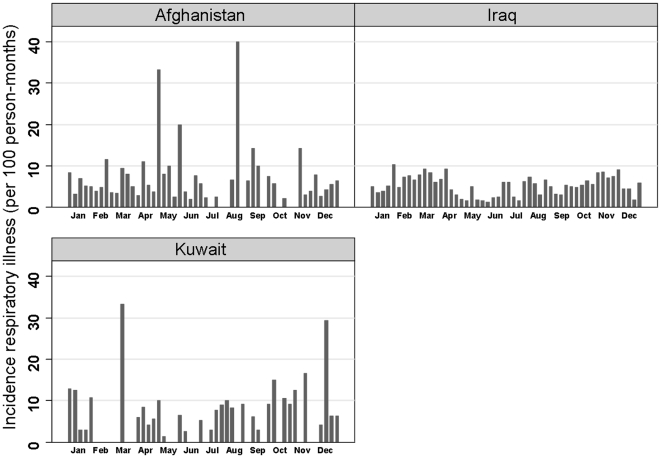
Probability of respiratory illness among U.S military personnel deployed in support of OIF/OEF by country of operation.

Overall rates of self-reported respiratory infection were 24.8 (95% CI 23.2–26.5) episodes per 100 person-months and were similar among those from Kuwait, Iraq and Afghanistan. (Data not shown). While overall rates were similar between these geographic regions, there were distinct differences in temporal patterns of disease noted. Weekly incidence of self-reported respiratory infection among troops in Iraq fell into a bimodal distribution with increased rates during the end of January to April and the latter part of September to December, respectively. The weekly incidence among troops in Kuwait had a similar bimodal distribution with significant increases during weeks 11 and 50. The distribution of weekly self-reported respiratory infection incidence among troops in Afghanistan did not have a similar pattern. Increased rates of respiratory infection were reported weeks 18, 22, 33, 36 and 45 during the period of May to November (Figure 1).

A multivariate negative binomial regression model for self-reported respiratory infection and covariates of age, gender, rank and country of deployment was fit. Similar to the deployment questionnaire analysis, increasing age and female sex remained independently associated with higher rates of self-reported respiratory infection. After adjusting for other factors, there remained a significant association between officers in the ranks O4–O6 (IRR 1.52, p = 0.048), as well as enlisted in the ranks E4–E6 (IRR 1.29, p = 0.04), and increased respiratory infection risk. After adjustment, there remained no significant association between increased respiratory rates and country of deployment (Table 4).

**Table 4:** Multivariate negative binomial regression of the association between rate of self-reported respiratory infection and covariates, Qatar Clinic health screening questionnaire Feb 2005 to Feb 2006. (n = 14,361).

Covariate	IRR	(95% CI)	P-value
Age (per year increase)	1.02	(1.0–1.03)	<0.001
Sex			
Male	Reference		
Female	1.44	(1.21–1.73)	<0.001
Rank			
E1–E3	Reference		
E4–E6	1.29	(1.02–1.62)	0.04
E7–E9	1.11	(0.76–1.63)	0.6
O1–O3	1.16	(0.78–1.73)	0.5
O4–O6	1.52	(1.00–2.32)	0.048
Chief Warrant Officer	1.50	(0.76–2.95)	0.2

## Discussion

As documented in previous studies, respiratory infections continue to be commonly reported among deployed military troops [4]. In our study, 40% reported having at least one respiratory infection while deployed, with a rate of 15.0 episodes per 100 person-months among those taking the voluntary deployment questionnaire and 24.8 episodes per 100 person months among those completing the clinic health screening form. Those taking the clinic form were asked to report any respiratory symptoms currently or in the past week. The higher reported rate of respiratory infection among those taking this questionnaire is likely due to less recall bias compared to voluntary questionnaire participants asked to report respiratory symptoms experienced over the course of the deployment (which averaged about 4 months). The overall percentage of troops deployed to OIF/OEF reporting respiratory infections, 40%, was also lower than that found in a previous study by Sanders et al. in which 69% of troops deployed to OIF/OEF from January to March 2004 reported having at least one respiratory infection [4]. Potential reasons for this decrease may be related to improved living conditions and facilities, semi-acquired immunity among those military personnel who participated on multiple deployments, increased seasonal vaccination rates against common respiratory viruses and better medical treatment, thus lowering the spread of infectious respiratory diseases among housed personnel. In addition, a previous study demonstrated that improved hand washing resulted in significantly lower rates of respiratory infection [1]. In this study, 69% of our population reported using flush toilets while 13% of the population in the Sanders, et al. study reported having access to a flush toilet [4]. This improvement of latrine facilities is likely linked to an improvement in hand washing facilities for troops in Iraq and Afghanistan, and may explain the lower percentage of self-reported respiratory infections in our study compared to that found by Sanders, et al. [4]. This relation in increasing risk of respiratory infection and decreasing level of sanitary hygiene, re-emphasizes the importance of field environmental preventive measures.

The negative impact of respiratory infections on individual and unit performance has been documented in previous studies [4]–[6]. Of concern is the apparent temporal increase in the percentage of deployed personnel that reported a decrease in job performance due to a respiratory infection in this study. Our results show 33.8% reported a decrease in job performance due to a respiratory infection compared to previous studies by Sanders, et al. and Paparello, et al., which found 14.1% and 7.4%, respectively [4], [5]. A possible reason for this increase may be related to increased job demand on deployed troops; therefore, even minor illness may have a larger impact on performance. It is also possible that personnel shortages and multiple deployments have increased cumulative demand and stress on troops, resulting in decreased performance or perceived decrease in performance with illness. Another possible reason for the increase in percentage of personnel reporting a decrease in job performance related to respiratory infections is that symptoms experienced may actually be more severe, resulting in a greater effect on job performance. However, percentage of personnel seeking medical care for respiratory symptoms has remained consistent with previous studies [4].

Increasing age was significantly associated with increased rates of self-reported respiratory infection. This remained significant after adjusting for confounding variables in analysis of the deployment questionnaire and health screening form. A previous study of respiratory disease in Saudi Arabia during Operation Desert Shield (ODS) revealed no association with rank or age [11]. However, the significant association found in our study may be due to differences between Operation Desert Shield and OIF/OEF in military mission, living conditions, length of deployment, location of deployment among other factors. This previous study found that less exposure to the outdoor environment was associated with respiratory symptoms such as sore throat and cough [11]. This may be an explanation for the higher rates of respiratory infection among senior enlisted and officers compared to junior enlisted, given usual differences in job description. Another possible reason may be increased recall and reporting among those with higher rank.

In this study, Navy personnel reported significantly higher rates of respiratory infection than all other military branches. This association remained significant in the multivariate analysis, even after adjusting for the other covariates. A previous study found that 78.7% of responding Navy personnel aboard the USNS Mercy during Operation Desert Shield reported at least one respiratory infection [5]. The rate of respiratory infection aboard ship was higher than that of ground troops stationed in Saudi Arabia during the same military operation [11]. Relatively small working and living spaces aboard ship were reported as main reasons for increased rates of infection in the previous study [5]. The Navy personnel in our study were unlikely to be shipboard, but rather primarily in the area of medical support personnel for the Marines. It is probable that the increased rate among the Navy was due to the health-care occupational exposures of managing respiratory infections among the troops. Further study of living and working conditions of all branches of the military may provide similar explanations for our results.

This study is unique as it allowed for broad sampling of U.S. troops deployed throughout CENTCOM AOR, as individuals left theater on R&R or upon redeployment over a one-year period. Responding military personnel in three sites, Kuwait, Qatar and Turkey, also allowed for capture of a more representative sample population. Mid- and post-deployment questionnaire of this large population of deployed troops also allowed detailed assessment of self-reported respiratory infection, perceived impact on performance, and associated risk factors and demographics. Access to two data sets from similar populations but different sources allowed for comparison and confirmation of findings. Because the voluntary questionnaire was anonymous, participants may have been more likely to answer truthfully, potentially providing a better estimate of self-reported respiratory infection and risk factors among deployed troops. In addition, the questionnaire form was short and easy to complete, resulting in 85% of questionnaires with all questions answered. The questionnaire also assessed demographics and details regarding respiratory infections among deployed troops, which had not been done previously during Operations Iraqi and Enduring Freedom.

There are several potential limitations of this study. Because our sample size was large, the study was potentially overpowered, leading to significance in results that may not otherwise be significant. Due to the nature of these questionnaires, self-reported data, medical diagnosis and infectious etiologies could not be assessed, which limits the information we have on respiratory pathogens among deployed troops. In addition, the questionnaire did not distinguish between rates of mild cold-like illness compared to more severe respiratory infections (e.g., pneumonia), although severity of illness was assessed indirectly through questions on fever, hospitalization, light duty/confined to quarters.

There is also the possibility of recall bias, since those with prior medical history or respiratory infections may have been more likely to recall illness or performance impact as compared to those who did not report respiratory disease. Selection bias may have also occurred, especially related to the use of convenience sampling and volunteers. Rates of severe respiratory infections requiring hospitalization may be underestimated due to selection bias. Those with severe infections may have been medically evacuated from theater and would not be available to participate in our study. In addition, there may be certain ranks, branches and types of units that may be under-represented due to fewer opportunities to participate in the R&R program, such as commanders and Special Operations personnel.

There are several future directions of research based on the results of this study. Further study is needed to explain the increased rates of respiratory infection particularly among females and Navy personnel. Additional research is also required to explore respiratory rate differences among age groups, ranks and locations of deployment. Assessment of variables such as living and working conditions, environmental factors and prior history of respiratory disease may provide more information. Linking a questionnaire study to a clinical or laboratory evaluation of respiratory pathogens would provide further information on diagnoses and etiologies of respiratory infection, as well as epidemiological data on respiratory pathogens among deployed troops. Surveillance and knowledge of common pathogens may also contribute to both the administration of existing vaccines and the development of novel vaccines, as well as encourage research on rapid diagnostic tests for use in a field environment. It is also important to further study the large increase in percentage of personnel reporting a decrease in job performance due to respiratory infection. Clinical and laboratory information would provide further information on severity of the reported respiratory infections and may provide explanations for decreased individual performance. Further evaluation of job demand and deployment stress, as well as the effects of multiple deployments on health and job performance is necessary. The U.S. military is currently involved in critical and challenging operations. The results of our study and these future areas of research will add to the military's medical capabilities, ultimately resulting in conservation of the fighting force.

## Methods

### Study Population and Data Sources

This descriptive study used self-reported questionnaire data from two sources collected from deployed troops in the Central Command Area of Responsibility (CENTCOM AOR) including, but not limited to Iraq, Afghanistan and Kuwait. A four day Rest and Recuperation (R&R) program was established for deployed U.S. military personnel [12]. Camp As Sayliyah in Doha, Qatar is one of the primary R&R locations. U.S. military personnel on mid-deployment R&R in Doha, Qatar and during redeployment from Camp Arifjan, Kuwait and Incirlik Air Base, Turkey between January 2005 and January 2006 were eligible to participate in the self-report mid- and post-deployment questionnaire. The questionnaire assessed self-reported respiratory illness as part of the Naval Medical Research Unit, No. 3 (NAMRU-3) Military Infectious Disease and Operational Health Surveillance Network. The questionnaire asked a series of demographic questions as well as ten questions related to respiratory illness. Informed consent was not required for the voluntary and anonymous questionnaire. Military personnel were asked if they would complete the questionnaire as part of their end-of-deployment rotation or their R&R check-in procedure. Researchers distributed the questionnaires to willing participants and collected the forms upon completion.

The second source of data were obtained as part of standard check-in procedures, among military personnel granted a mid-deployment R&R who were required to complete a clinic health questionnaire screening form, reporting any current or recent health problems that may require medical attention. Among screening for a variety of illnesses and injuries, the questionnaire asked currently or in the last week have you had, “Any respiratory infection (e.g. cold, cough, sore throat)?,” “Any fever?,” as well as demographic variables to include military unit, age, gender, rank, location of deployment and current medications.

### Data Analysis

Descriptive analyses were conducted on all data using standard statistical methodologies. Incidence estimates for overall respiratory illness, clinic-visit associated respiratory illness, confinement to quarters and hospitalization (associated with respiratory illness) were calculated based on self-reported events and person-time in theater using negative binomial regression (Poisson regression analysis assumptions not met). The associations between respiratory illness and possible associated variables were initially explored by univariate negative binomial regression methods. Multivariable negative binomial regression models were used to evaluate the relationship between respiratory illnesses and variables found significant on univariate analysis while adjusting for potential confounding. Using a backwards elimination approach to avoid underfitting the models, all variables were initially added to the models. The variable with the largest insignificant p-value was removed, and the models were re-fit. This process was continued iteratively until all variables retained in the models were significant at the alpha = 0.15 level.

Data was double-entered into MS Access (Microsoft, Inc., Redmond, WA) and Stata V9 (StataCorp LC, College Station, TX) was used for all data analyses. Statistical significance was two-tailed and set at p<0.05.

### Ethics Statement

The NAMRU-3 IRB, Cairo, Egypt, approved the original data collection using the self-report deployment health questionnaire, under protocol DoD# NAMRU3.2005.0012. The de-identified data obtained from the Troop Medical Clinic Health Screening Questionnaire were collected for a primary clinical purpose. The NAMRU-3 IRB reviewed our protocol and categorized this work as exempt.
